# The crowding dynamics of the motor protein kinesin-II

**DOI:** 10.1371/journal.pone.0228930

**Published:** 2020-02-13

**Authors:** Vandana S. Kushwaha, Seyda Acar, Daniël M. Miedema, Dmitry V. Denisov, Peter Schall, Erwin J. G. Peterman

**Affiliations:** 1 Department of Physics and Astronomy and LaserLaB, Vrije Universiteit, Amsterdam, Netherlands; 2 Institute of Physics, University of Amsterdam, Amsterdam, Netherlands; 3 Laboratory for Experimental Oncology and Radiobiology (LEXOR), Center for Experimental Molecular Medicine (CEMM), Academic Medical Center, Amsterdam, Netherlands; Tata Institute of Fundamental Research, INDIA

## Abstract

Intraflagellar transport (IFT) in *C*. *elegans* chemosensory cilia is an example of functional coordination and cooperation of two motor proteins with distinct motility properties operating together in large groups to transport cargoes: a fast and processive homodimeric kinesin-2, OSM-3, and a slow and less processive heterotrimeric kinesin-2, kinesin-II. To study the mechanism of the collective dynamics of kinesin-II of *C*. *elegans* cilia in an *in vitro* system, we used Total Internal Reflection Fluorescence microscopy to image the motility of truncated, heterodimeric kinesin-II constructs at high motor densities. Using an analysis technique based on correlation of the fluorescence intensities, we extracted quantitative motor parameters, such as motor density, velocity and average run length, from the image. Our experiments and analyses show that kinesin-II motility parameters are far less affected by (self) crowding than OSM-3. Our observations are supported by numerical calculations based on the TASEP-LK model (Totally Asymmetric Simple Exclusion Process-Langmuir Kinetics). From a comparison of data and modelling of OSM-3 and kinesin-II, a general picture emerges of the collective dynamics of the kinesin motors driving IFT in *C*. *elegans* chemosensory cilia and the way the motors deal with crowding.

## Introduction

Differentiated and specialized cells generate specific transport needs and encounter a variety of transport challenges. Transport along cytoskeletal filaments is driven by members of the kinesin, dynein and myosin superfamilies of motor proteins, often engaged in teams and highly regulated. Unraveling the collective dynamics of different motor species is very important to understand how intracellular transport can run smoothly in a cellular environment. A key example of collective and collaborative transport by multiple, densely packed motor proteins is intraflagellar transport (IFT) driven in the anterograde direction by kinesin-2 motors. In the chemosensory cilia of *C*. *elegans*, teams of two Kinesin-2 motors: ~50 slow heterotrimeric kinesin-II (KLP-11/20 KAP-1) and ~30 fast homodimeric OSM-3 teams, work together to drive transport of IFT trains from ciliary base to tip. Kinesin-II is mostly active in base and transition zone, after which OSM-3 gradually takes over to drive transport towards the tip [1]. This functional specialization most likely finds its origin in distinct motility properties of the two motors, including velocity, run length and ability to circumvent obstacles on microtubules [1]. In previous *in vitro* studies, it has been demonstrated that the motility properties of conventional kinesin (kinesin-1) are negatively affected by static [2, 3] and dynamic crowders [4–7], situations that are likely to occur in the cell. Also, other kinesin motor proteins like kinesin-8 [8] have been shown to slow down at high motor densities, just like cars in a traffic jam. Recently [7], we employed a combination of high motor density motility assays, image correlation analysis and simulations on kinesin-1 (from *Drosophila melanogaster*) and OSM-3 (homodimeric kinesin-2 from *C*. *elegans*). We found that the kinesin-1 motors start affecting their velocity and run length at motor densities above ~2 motors/μm and become noticeable with effective interaction length 60 nm, while OSM-3 motors only start affecting each other at much higher densities of ~30 motors/μm, implying vanishing effective interaction length. In the current study, we apply similar methods to a kinesin-II construct consisting of truncated versions of the KLP-11 and KLP-20 proteins combined with the tail of kinesin-1 for proper bacterial expression and dimerization. Kinesin-II’s function *in vivo* is to transport, in large groups, cargo through the densely packed transition zone, which might require different motility behavior under crowding conditions than OSM-3 and kinesin-1.

Heterotrimeric kinesin-2 motors, kinesin-II in *C*. *elegans*, KRP85/95 in sea urchin and KIF3A/B in mammals, are intriguing members of the kinesin super family, with the particular property of having two distinct (instead of identical) motor domains, at which ATP is hydrolyzed and the motor binds to a microtubule. Heterotrimeric kinesin-2 motors typically contain a third polypeptide, Kinesin-Associated Polypeptide (KAP), without motor function [9,10], which interacts with the tail domains, facilitates and regulates motor activity [11,12]. Heterotrimeric kinesin-2 has been shown to generate torque and axial force to a microtubule [13–16]. Furthermore, it has been shown that KIF3A/B is relatively good in navigating statically crowded microtubules, most likely by side stepping [17].

Here, our goal is to unravel the motility behavior of *C*. *elegans* kinesin-II under highly crowded conditions, mimicking traffic jams, using a combination of high-density motility assays, image correlation analysis and simulations on the Totally Asymmetric Simple Exclusion Process with Langmuir Kinetics-(TASEP-LK; [7]). We find that kinesin-II is substantially less affected by self-crowding than other kinesin tested, Kinesin-1 and OSM-3 [7].

## Materials and methods

Unless mentioned otherwise, all chemicals were purchased from Sigma-Aldrich. All concentrations given are final concentrations.

### Microtubules preparation

Microtubules were prepared from in-house purified bovine brain tubulin similar to as described previously [18]. Tubulin (5 mg/ml) in PEM80 [80mM PIPES (Piperazine-N,N′-bis(2-Ethanesulfonic acid) pH 6.9, 1 mM EGTA (Ethylene-Glycol-Tetraacetic Acid), 2mM MgCl_2_ (Fluka analytical 63072)] buffer supplemented with 4 mM MgCl_2_, 1 mM GMPCPP (non-hydrolysable GTP-analogue, Jena Bioscience NU-405S) in 5% (v/v) DMSO (Dimethyl Sulfoxide) was incubated for 30 min at 37°C to polymerize. Microtubules were stabilized by mixing 1:20 (v/v) with PEM 80 containing 10μM Taxol. Microtubule preparations were used within a week [19].

### Cloning and preparation of motor proteins

The *C*. *elegans* heterotrimeric kinesin-II protein consists of three polypeptide chains: KLP11, KLP20 and KAP1. Here we created short, *E*. *coli* expressible heterodimeric constructs of this protein, containing the motor domains and native neck linkers of KLP11 (1–357 amino acids) and KLP20 (1–345 amino acids), connected by the stable and well-characterized *Drosophilla melanogaster* Kinesin-1 stalk (88 amino acids). To this end, we constructed the a klp-11 construct with a superfolder GFP (sfGFP) on the 3’end of the Kinesin-1 stalk. The construct contained an 8 amino acid long Strep-tag for purification. The klp-20 construct was cloned in a similar way, but with a SNAP-tag^®^ (NEB) and a 6x His-tag for affinity purification. These two constructs were cloned into the two multiple cloning sites of a pETDuet-1 vector (Novagen). Plasmids containing double inserts were transformed into Rosetta^™^ 2(DE3) competent *E*. *coli* cells (Novagen). Protein expression was induced by addition of 0.4 mM IPTG to a total of 1.6 L culture (4x400 mL each) in 1 L Erlenmeyer flasks. Expression was allowed to proceed for 6 hours at 22°C, 220 rpm in a shaker incubator (Infors HT-Ecotron). Heterodimeric kinesin-II protein was purified using two-step affinity purification; a first step using Strep-Tactin^®^ (IBA Life Sciences) affinity chromatography, followed by a step using Ni-NTA resin. The purified protein shows two distinct bands in SDS PAGE analysis, corresponding to the molecular weight of expressed KLP-11 and KLP-20 constructs.

In addition, a non-labeled version of this heterodimeric kinesin construct was created by simply replacing the sfGFP gene with a Snap-tag in the klp-11 construct. The plasmids of unlabeled heterodimer kinesin-II was transformed, expressed and purified as stated for the GFP-labeled protein.

The concentration of the purified unlabeled kinesin-II and sfGFP-labeled kinesin-II constructs were determined using Bradford assays with bovine serum albumin as standard [20] to be 0.29 mg/ml and 1 mg/ml, respectively. Freshly purified proteins were aliquoted in 10 μl vials in PEM80 buffer containing 20% (v/v) sucrose, and snap frozen immediately in liquid nitrogen and stored at -80°C until use.

### Kinesin-II-microtubule crowding assay

Assays were prepared in hydrophobic, silanized and cleaned flow cells (volume ~5–8 μl), constructed from a microscope slide (Menzel-Gläser, 76×26 mm, cut edges) attached to a coverslip (High Precision, Deckgläser, 22x22 mm, thickness 170±5 μm) with double-sided Scotch tape as a spacer. A detailed description of the glass surface preparation is provided in in Appendix A of our previous study [7].

To immobilize microtubules, the sample chambers were first incubated with 2 μg/ml monoclonal anti-β-tubulin antibody (Sigma T7816) in PEM 80 with 10 μM Taxol (hereafter denoted as PEM80T). After 5 minutes incubation, excess antibodies were flushed out with PEM80T, followed by 15 minutes incubation with 1% (w/v) Pluronic F-127 in PEM80T. Excess Pluronic F127 was flushed out by rinsing chambers with PEM80T. Finally, sample chambers were incubated for 10 minutes with 0.1 mg/ml microtubules in PEM80 with 10 μM Taxol to allow them to attach to the surface via the antibodies. After attaching the microtubules, the sample chambers were flushed with PEM12 buffer (12 mM PIPES (Piperazine-N,N′-bis(2-Ethanesulfonic acid) pH 6.9, 1 mM EGTA, 2mM MgCl_2_). In the final step, the sample chambers were flushed with motility solution supplemented with 10 μM Taxol, 2 mM additional MgCl_2_, 2 mM ATP (Adenosine 5’ TriPhosphate), 0.2 mg/ml casein in PEM80, an oxygen scavenging cocktail containing 200 μg/ml glucose oxidase, 72 μg/ml catalase, 22.5 mM glucose, and 10 mM DTT (dithiothreitol) and kinesin-II (with a final concentration varying from 5 nM to 1250 nM). In the crowding motility experiments, the concentration of fluorescent sfGFP-kinesin-II was kept constant at 5 nM and the concentration of unlabeled kinesin-II was increased resulting in ratios of 1:0, 1:10, 1:20, and 1:40, 1:100 and 1:250. Finally, sample chambers were sealed with VALAP (1:1:1 vaseline, lanoline, paraffin). Experiments were performed in both PEM12 (12 mM PIPES pH 6.9, 1 mM EGTA, 2mM MgCl_2_) and PEM 80 (80 mM PIPES pH 6.9, 1 mM EGTA, 2mM MgCl_2_) buffers. One thousand images were acquired continuously with a 200-ms exposure time without delay in between. Multiple microtubules (2–4) were analyzed per image stack, adding up to a total of ~70 analyzed microtubules in PEM12 buffer and ~35 analyzed microtubules in PEM80 buffer.

### Image acquisition and data analysis

#### Instrumentation

Experiments were performed at 23°C using Total Internal Reflection Fluorescence (TIRF) microscopy. Microscopy images were acquired using a custom-built TIRF microscope operated by the Micro-Manager software interface (μManager, Micro-Manager1.4, https://www.micro-manager.org/), built around an inverted microscope body (Nikon, Eclipse Ti) fitted with a 100×oil-immersion objective (Nikon, CFI Apo TIRF 100X, N.A. 1.49). Excitation light, provided by a diode-pumped solid-state laser (Cobolt Calypso 50TM 491 nm DPSS), was first passed through an AOTF (AA Opto-Electronics, AOTFnC-400.650-TN) for wavelength selection, next through a quarter wave-plate (Thorlabs, mounted achromatic quarter-wave plate, 400–800 nm, AQWP05M-600) to obtain circularly polarized light, and a dichroic mirror (Semrock, 405/488/561/635 nm lasers Brightline® quad-edge laser-flat, Di 03-R405/488/561/635-25x36) was used. The laser intensity was ~81 W/cm^2^ in the image plane. Emission light was separated inside the Optosplit III using a dichroic long pass filter. Images were acquired by an EMCCD camera (Andor, iXon DU-897E-COO-#BV). The camera has a pixel size of 16x16 μm, and with a 200x magnification this results in an effective pixel size of 80x80 nm. Assays were mounted on microscopic stage and after 3 minutes, images were acquired continuously using an exposure time of 200 ms without any delay and saved as 16-bit tiff files. Special care was taken to minimize the effects of photobleaching and other potential sources of artifacts (such as background noise and inactive motors) by optimizing time resolution and duration of the experiments [7].

#### Single particle tracking

For single-particle tracking, a sequence of 1000 fluorescent images were analyzed using custom-written routines in MATLAB (The MathWorks, Natick, MA), a modified version of the tracking algorithm utrack [21,22] Robust single-particle tracking in live-cell time-lapse sequences. First, fluorescent molecules were detected for each image. The location and intensity of the individual fluorescent molecules were obtained by a two-dimensional Gaussian fit. The localized particles in subsequent image frames were linked to obtain single-particle trajectories using the utrack linking algorithm. For displaying purposes, kymographs (time-space plots) were generated from the image sequences using the ImageJ macro toolset *KymographClear* [23].

#### Correlation imaging-based image analysis

In order to extract motor density, average motor velocity and run length of fluorescently labeled kinesin-II motors at high densities, we used custom-written routines in MATLAB (MathWorks, Natick, MA) for automated image analysis based on image correlation [7]. First, the beginning and end of microtubule segments to be analyzed were selected and background was subtracted (Eq. B3 in [7]). The software then determined the intensity along the microtubule axis for all images in the stack I (x; t). The motor density on a microtubule segment was calculated using Fluorescence Correlation Spectroscopy (FCS) from the temporal intensity fluctuations (spatially averaged). For velocity and run length determination, the spatiotemporal correlation was calculated. By tracing the autocorrelation peak using Gaussian fitting, the average velocity v = dx/dt was determined (Fig 2D and 2E, [7]). From the temporal evolution of the area A under the curve, the run length could be estimated (Fig 2F, [7]).

#### TASEP-LK model

TASEP-LK (Totally Asymmetric Simple Exclusion Process with Langmuir Kinetics) simulations were performed as discussed in [7]. In brief: particles were allowed to bind to a microtubule track with rate ω_A_. They moved with constant velocity in one direction and detach with rate ω_D_. Particles were not allowed to pass each other: when the next binding site on the track was occupied, a particle stalled or detached with detachment rate ω_DC_. In our simulations, we allowed multiple, independent, parallel tracks (mimicking the protofilaments in a microtubule) [7]. We did not allow for sidestepping between the parallel tracks, since this would introduce additional parameters that are difficult to verify directly experimentally, but mainly because we focus here particularly on the high-density regime. We reasoned that in the high-density regime, side stepping hardly occurs, since in most cases track next to a motor under consideration would be occupied by another motor.

To arrive at simulation results comparable to the experimental results we followed the same staps as in [7]. First, we simulated using the standard TASEP-LK model. At low concentrations, density, run length and velocity depend linearly on concentration, allowing (by linearly fitting experimental data in this low-concentration regime) to extract affitnity, detachment rate ω_D_ and step frequency. Next, using these parameters, simulations can be run in the full concentration range, from low to very high density. In the medium to higher density regimes additional parameters need to be included: interaction range, detachment rate of constrained motors (ω_DC_) and number of lanes [7]. The number of lanes has been varied in the range of 1–13 (the maximum number of protofilaments per microtubule), the interaction range was varied in the (integer) range of 0 to 20 motor sizes, while ω_DC_ was varied in the (integer) range of 0–10 times ω_D_ to find a reasonable fit with experimental data.

## Results

### Probing the motility properties of purified kinesin-II at the single-molecule level

**Fig 1:**
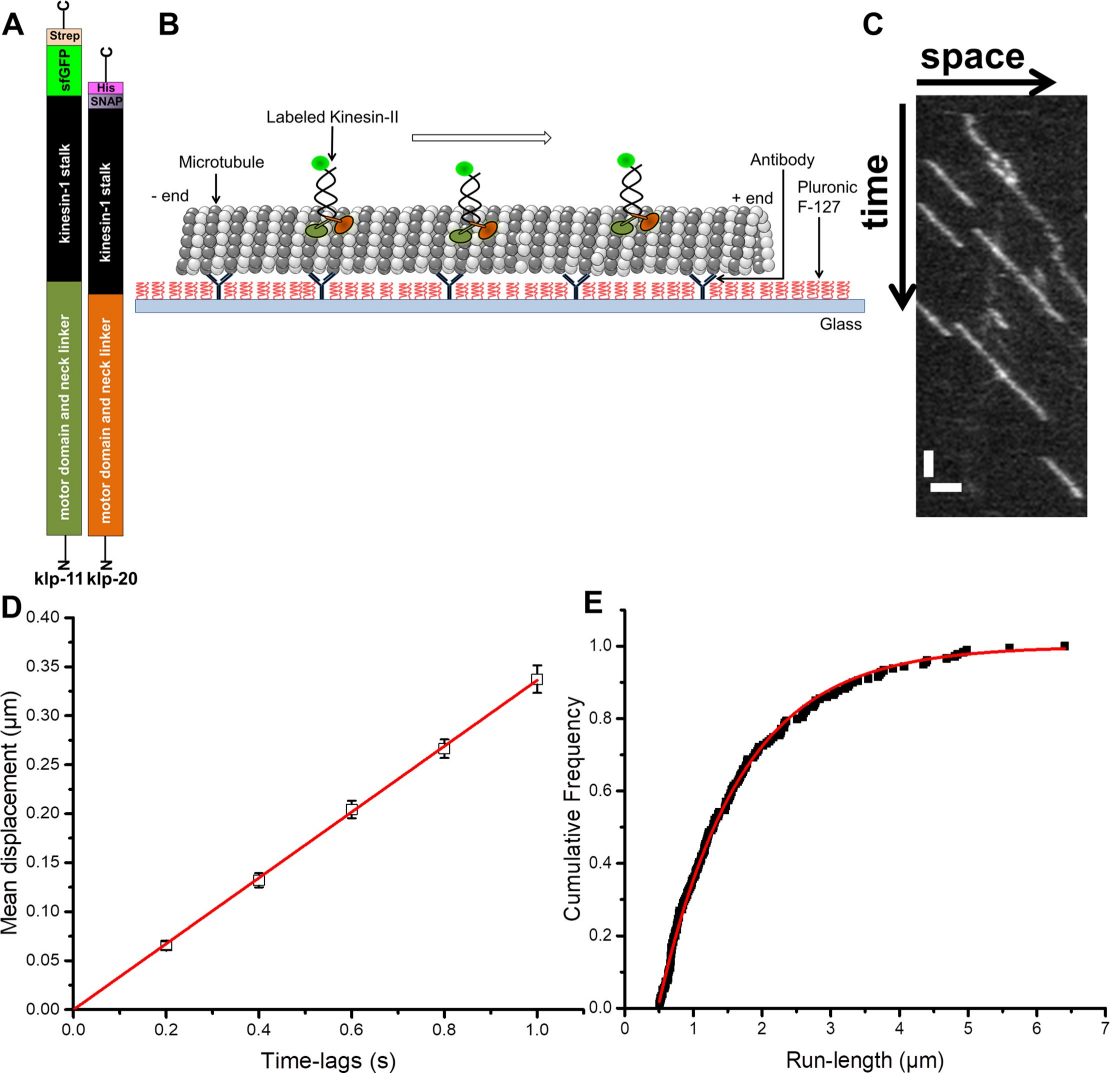
Motility properties of kinesin-II at the single-molecule level. A. Design of a labeled heterodimeric kinesin-II construct. B. Schematic of the *in vitro* motility assay. Single sfGFP-labeled kinesin-II molecules are observed moving along unlabeled, surface-immobilized microtubules. C. Typical kymograph (space-time plot) of individual sfGFP-kinesin-II motors obtained from single molecule TIRF motility assays. Time is progressing from top to bottom, length from left to right; scale bars: 1 μm (horizontal) and 2 s (vertical); the kinesin-II concentration was 200 pM. D. Mean displacement versus time-lag plot obtained from mean displacements extracted from 237 individual sfGFP-kinesin-II trajectories. Error bars indicate standard error of the mean (SEM). Red: linear fit with slope 0.33 ± 0.01 μm/s (R^2^ = 0.99). E. Cumulative probability distribution of lengths (μm) of individual trajectories. Red: exponential fit yielding an average run length of 1.18 ± 0.07 μm (R^2^ = 0.98).

To characterize the motility parameters of our heterodimeric sfGFP-kinesin-II construct, we performed single-molecule motility assays using Total Internal Reflection Fluorescence (TIRF) microscopy (Fig 1B). Our dimeric construct contains the two motor domains and neck linkers of *C*. *elegans* heterotrimeric kinesin-II (KLP11 and KLP20), the stalk of *Drosophila* kinesin-1, a sfGFP on one of the chains and affinity tags for proper purification of the heterodimeric construct (Fig 1A). This approach follows a procedure previously devised for the mamalian homologs of heterotrimeric kinesin-II, KIF3A/B [24]. Our reasoning to use this construct is that we needed a consitutively active version of Kinesin II (i.e. without functionality to auto-inhibit [13]) that could be expressed in *E*. *coli*. Motility experiments were performed at low-salt conditions using PEM12 buffer. Fig 1C shows a typical kymograph of individual sfGFP-kinesin-II motors stepping along microtubules, obtained from recorded image stacks. In the kymograph, single-motor trajectories can be discerned as slanted lines, with more or less constant slope, indicative of motion with constant velocity. The kinesin-II motility parameters were obtained by extracting single-motor trajectories from the time-series of TIRF images using Single-Particle Tracking (SPT). From the trajectories, the average mean-displacement was obtained for different time lags (Fig 1D). The data could be fitted with a straight line yielding the average velocity, 0.33 ± 0.01 μm/s. This velocity is slightly lower than the *in vivo* velocity in *C*. *elegans* chemosensitive neurons (~0.5 μm/s [1]) and comparable to studies of full-length *C*. *elegans* kinesin-II (KLP11/KLP20) *in vitro* using microtubule gliding assays (0.3 μm/s) [25] and single-motor walking assays (0.40 ± 0.06 μm/s) [15]. In addition, we determined the cumulative probability distribution of the length of individual trajectories (Fig 1E) and fitted that with an exponential function, yielding an average run length of 1.18 ± 0.07 μm. This run length is substantially shorter than what has been measured *in vitro* for the full-length construct (2.8 ± 0.5 μm) [15], which very likely is due to the shorter length of our constructs and the different buffer conditions used.

### Kinesin-II motility properties under crowded conditions using correlation-based image analysis

**Fig 2:**
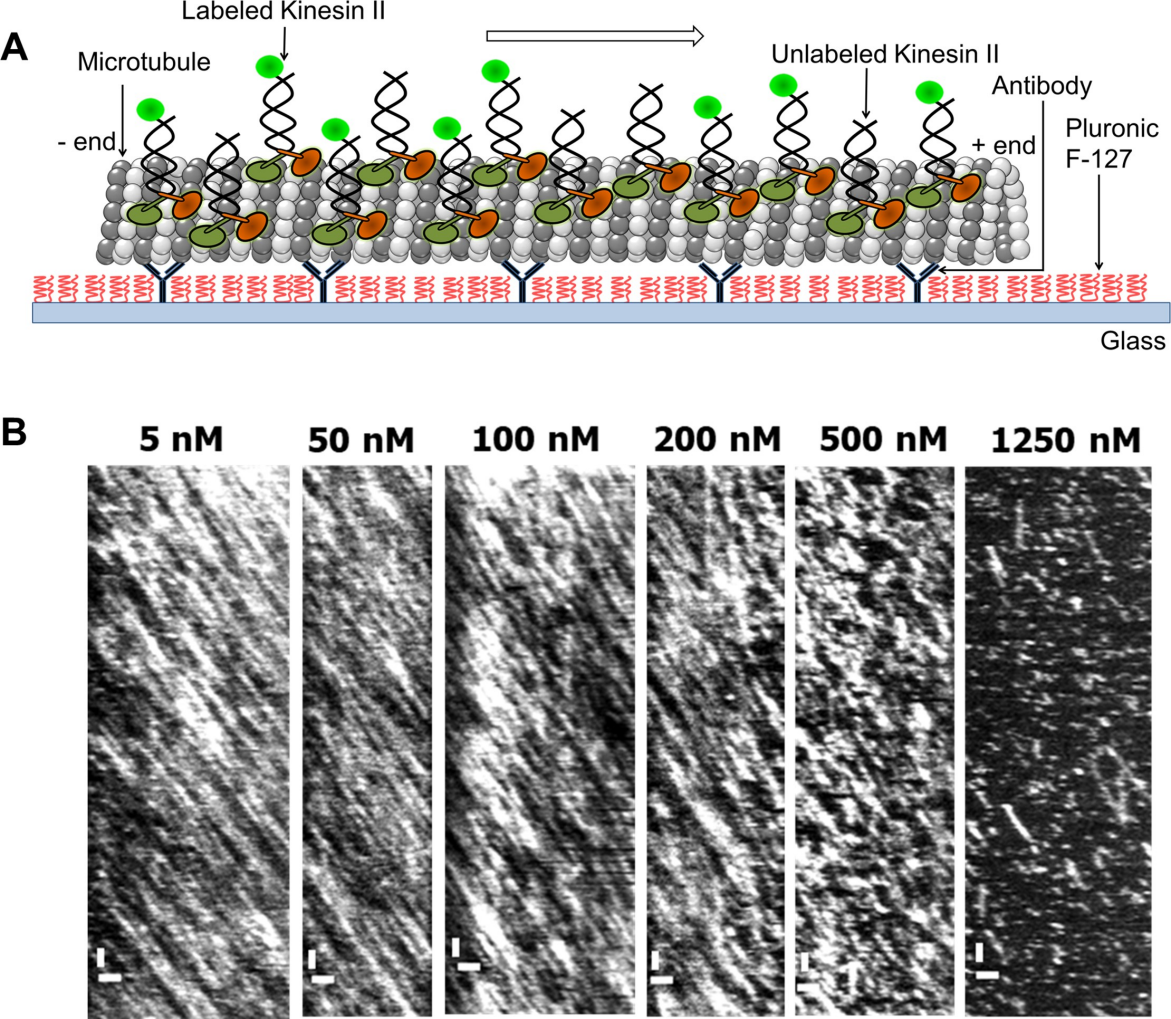
Kinesin-II motility under crowded conditions. A. Schematic of the *in vitro* motility assay. kinesin-II motors walk along the microtubules attached to the glass slide. The fluorescently labeled motor proteins are excited and imaged using TIRF microscopy. B. Kymographs (scale bars: 1 μm (horizontal) and 2 s (vertical)) of kinesin-II motility extracted from time series of TIRF images. The total concentration of labeled plus unlabeled motors is indicated. sfGFP-kinesin-II concentration is 5 nM in all kymographs. Measurements were performed in PEM12 buffer.

To study kinesin-II motility parameters under crowded conditions, we recorded time series of TIRF images using the same assay discussed above (in PEM12 buffer) but using higher concentrations of motors. To keep image conditions similar between measurements at different concentrations, we used a mixture of labeled sfGFP kinesin-II and unlabeled kinesin-II motors, keeping the concentration of labeled motors constant (5 nM) and varying the amount of unlabeled motors (from 0 to 1245 nM), as in previous studies [3,6,7], Fig 2A. Representative kymographs extracted from such data (at total motor concentration ranging from 5 nM to 1250 nM) are shown in Fig 2B. Kymographs clearly indicate that kinesin-II runs become shorter, while the velocity decreases at concentrations of 500 nM and above.

**Fig 3:**
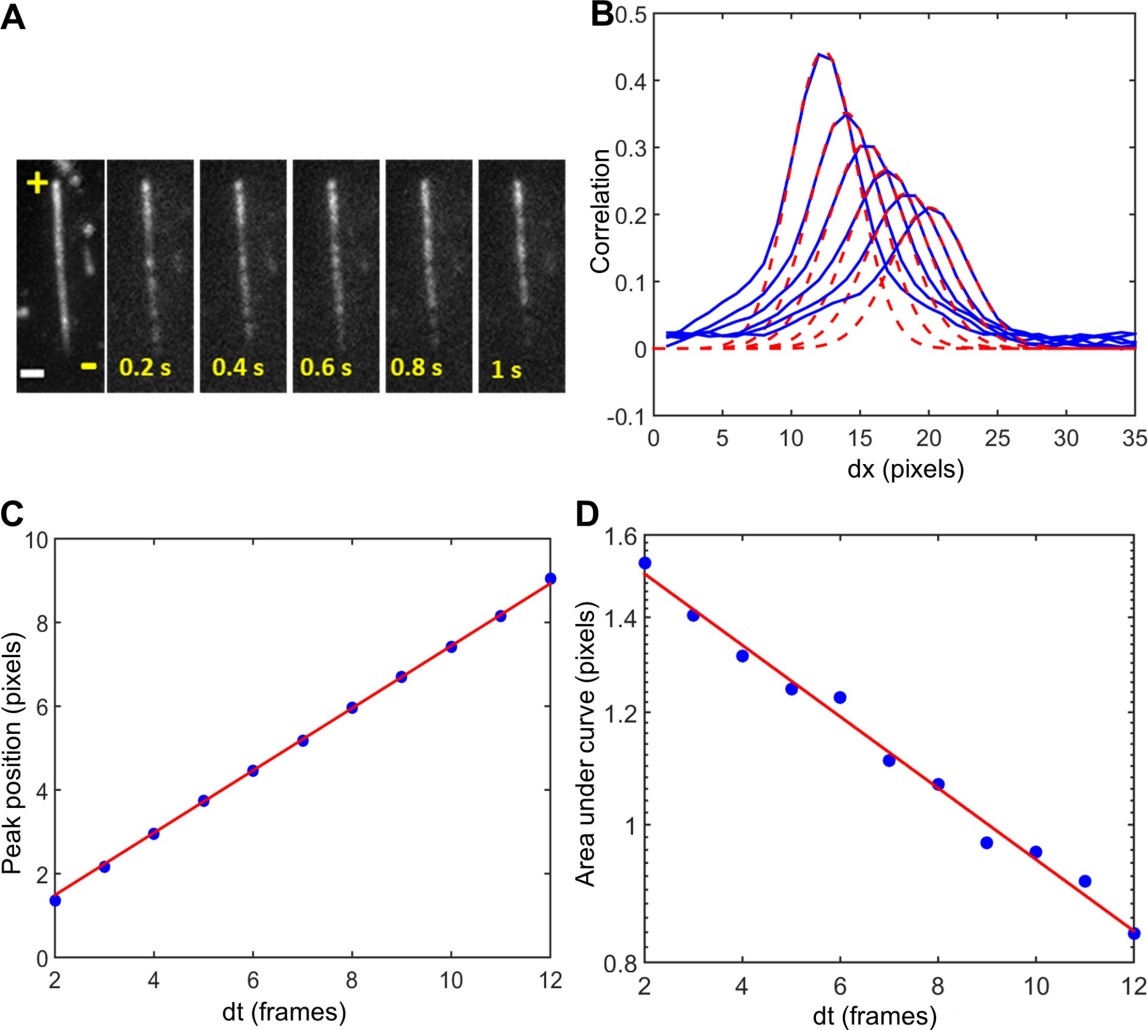
Correlation imaging measurements at high density of kinesin-II. A. An example of one of the experiments; time series of TIRF images of fluorescently labeled kinesin-II motor proteins on a microtubule at a concentration of 5 nM in PEM12 buffer, exposure time 0.2 s, 1 pixel corresponds to 0.08 μm. The plus and minus ends of the microtubule are indicated. B. Cross sections of the correlation surface at different time lags. Gaussian fits (dashed red line) are used to obtain the peak position and area under these curves. C. Peak position as a function of time. The red linear fit yields the motor velocity of 0.30 ± 0.02 μm/s, R^2^ = 0.99 D. Area under the curve as a function of time in semi-logarithmic representation. An exponential fit (red) yields the detachment rate of motors, from which the average run length can be determined to be 1.2 ± 0.3 μm, R^2^ = 0.96.

The kymographs are substantially more crowded than those at single-molecule conditions (Fig 1), indicating that images cannot be analyzed using single-particle tracking. To extract motility parameters (motor density, velocity and run length), we resorted to image correlation analysis (Appendix B, [7]). To determine motor density, we calculated the time correlation of intensity fluctuations on microtubules, similar to Fluorescence Correlation Spectroscopy (FCS). To determine motor velocity and run length, image intensities of the time series of TIRF images were correlated in space and time along a microtubule x-axis, pixel by pixel. The peak of the autocorrelation for different time lags was determined by Gaussian fitting (Fig 3A and 3B). The time dependence of the position of the correlation maximum was used to determine the average motor velocity (Fig 3C) and the area under the autocorrelation to determine the average run length (Fig 3D). The extracted quantitative results for velocity and run length from correlation imaging are in excellent agreement with the single-particle tracking results at low density (Fig 1).

### Kinesin-II density increases linearly with concentration, up to high concentrations

**Fig 4:**
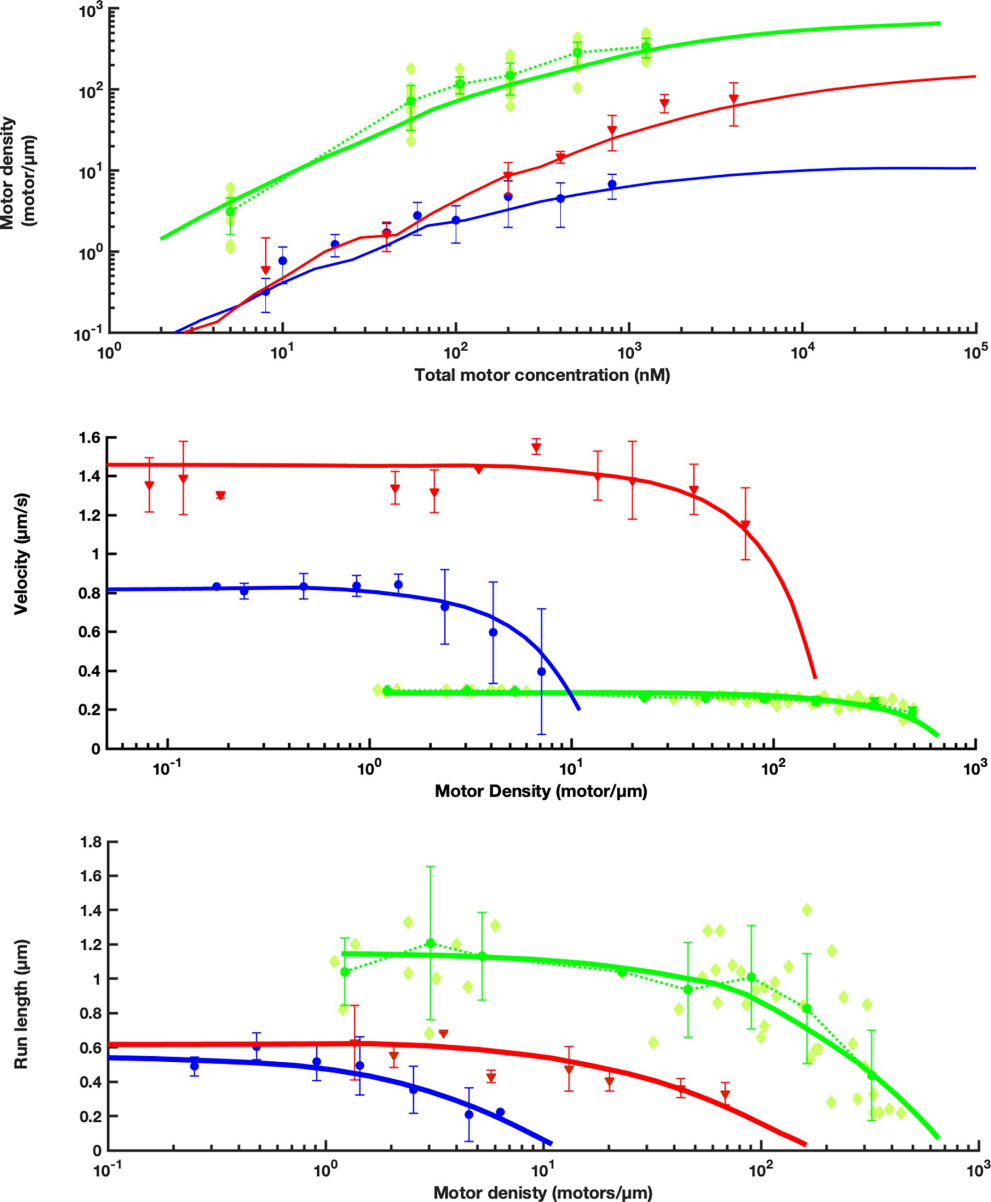
**Kinesin-II motility parameters as a function of total motor concentration (green), compared to previously published data and simulations from kinesin-1 (blue) and OSM-3 (red) [7].** A. Motor density (total density of labeled and unlabeled motor proteins) on microtubules as function of total motor concentration, calculated from fluorescence intensity fluctuations on ~70 microtubule segments. Light green diamonds: data points of individual microtubule segments (kinesin-II); symbols with error bars: averages and standard deviations calculated at each concentration; curves: predictions of the extended TASEP-LK model. B. Motor velocity as a function of motor density on microtubules. Light green diamonds: velocity determinations on individual microtubule segments (kinesin-II); symbols with error bars: averages and standard deviations of multiple velocity determinations within logarithmically scaled density intervals; curves: predictions of the extended TASEP-LK model. C. Run length as a function of motor density on microtubules. Light green diamonds: run length determinations on individual microtubule segments; symbols with error bars: averages and standard deviations of multiple run length determinations within logarithmically scaled density intervals; curves: predictions of the extended TASEP-LK model.

In a first analysis of the data, we focused on the density of motors on a microtubule as a function of motor concentration in solution, as obtained from temporal correlation of the fluorescence intensity, Fig 4A. One would expect motor density to scale linearly with concentration, until the binding sites on a microtubule start to become saturated. Qualitatively, that is indeed what we observed. Up to a concentration of ~100 nM, the density roughly scales linearly with concentration. At higher concentrations, saturation of the binding sites results in a decrease of the slope of the curve. The experimental data show that the maximal density we obtained was ~400 motors/μm. Assuming that each motor protein occupies 16 nm, one microtubule protofilament could fit 62 motors/μm, a complete 13-protofilament microtubule ~800 motors/μm, but not all of the protofilaments are expected to be accessible, due to the surface attachment. This indicates that, in our PEM12 experiments, we reached substantial saturation of the microtubule surface with kinesin-II. Under higher salt conditions (PEM80), the affinity of kinesin-II for microtubules was substantially lower and no sign of saturation of binding sites could be observed for the motor concentrations tested (S1 Fig).

### Kinesin-II velocity decreases only at very high density

We next focused on the velocity of kinesin-II as a function of motor density, obtained from correlation analysis of the images (Fig 4B). At low motor densities (<10 motors/μm), the velocity was 0.30 ± 0.02 μm/s, very similar to the velocity obtained above from single-particle tracking. At higher motor densities, the velocity decreased, most likely due to ‘traffic jams’ caused by motors that are not able to proceed stepping forward, since the next binding site on the microtubule is occupied by another motor. We note that the velocity decrease is only modest: at a very high density of ~400 motors/μm, where at least 50% of all potential binding sites on a microtubule are occupied, the velocity has only dropped by a third. A 50% drop would have been expected in case the occupation of the microtubule would be uncorrelated.

### Run length of kinesin-II motors changes with density

In this section, we focus on the average run length of kinesin-II as a function of motor density, obtained from correlation analysis of the images Fig 4C. At the lowest densities, <10 motors/μm, the average run length was 1.2 ± 0.3 μm, the same as obtained from single-particle tracking. At densities above ~200 motors/μm, the average run length decreased faster than velocity, the faster decaying run length indicates a density-dependent binding time. At the highest density obtained (~400 motors/μm), the run length had decreased ~5-fold. Under these higher salt conditions (PEM80), no motor densities could be obtained that were high enough to cause crowding effects in the average run length.

### TASEP-LK modelling

Next, to obtain further insights in our data and understand kinesin-II crowding dynamics, we applied the TASEP-LK modelling scheme we used before for studying motor crowding [7]. In this approach, we assumed that motors move independently along lanes (microtubule protofilaments) and that sidestepping does not occur. For a motor to step forward, the next binding site on the lane has to be empty. When the next binding site is occupied by another motor, the motor under consideration pauses or detaches. The detachment rate of a paused motor is allowed to be larger than that of an unconstrained motor. We found that we obtain best fits to the experimental data when the detachment rate of constrained motors is 3 times as high as that of unconstrained motor. We further found that the number of lanes is 13, which suggests the microtubule is fully occupied which could be attributed to motor domain specific biophysical and biochemical properties. Under these conditions, a reasonable description of the experimental kinesin-II density, velocity and run length under crowding can be obtained using the further parameters given in Table 1.

**Table 1:** Fitting and derived parameters for the modified TASEP-LK model for kinesin-II in PEM12 buffer.

Quantitative Parameters	Kinesin-II PEM12
Motor size, nm	16
Motor step, nm	8
Affinity (ω_A0_), μm^3^/sec	1.53x10^-4^
Detachment rate (ω_D_), s^-1^	0.25
Detachment rate constrained motors (ω_DC_), s^-1^ (ω_DC_ = 3ω_D_)	0.75
Step frequency, s^-1^	37.5
Number of lanes	13
Interaction range, nm	0
Velocity at ρ →0, μm/sec	0.3
Run length at ρ →0, μm	1.14

The Motor size is the length a motor occupies on a single microtubule-protofilament. The Affinity is determined by dividing motor density on microtubules and motor concentration in solution in the low-density regime. The Detachment rate is obtained by dividing the velocity by the run length of motors in the low-density regime. Step frequency is the number of steps per second (velocity divided by Motor step). Number of lanes is the number of protofilaments in a microtubule accessible for the motors.

## Discussion

In this study, we explored the effect of kinesin-II crowding on kinesin-II density on the microtubule, velocity and run length, using *in vitro* crowding motility assays. Our results indicate that heterodimeric kinesin-II is far less affected by motor crowding than kinesin-1 and OSM-3 [7]. We infer that this shows that the motility parameters of kinesin-II are specifically adapted to work efficiently in highly crowded conditions, such as those found in cilia, where this motor drives intraflagellar transport.

Before, we performed similar crowding studies to kinesin-1 from *Drosophila* and OSM-3, a homodimeric kinesin-2, also involved in *C*. *elegans* intraflagellar transport (for comparison with current results, data also included in Fig 4). Like for kinesin-II, for these motor proteins the motor density on microtubules scaled linearly with motor concentration at low concentrations, until saturation. Velocity and run length of all three motors were relatively independent of density at low motor concentrations but dropped dramatically at higher densities. We could model this behavior using a TASEP-LK model assuming increased detachment rates of jammed motors. Although the qualitative behavior of the two motor proteins was similar, remarkable quantitative differences to the current motor kinesin II were observed, in particular with respect to the minimum motor density at which velocity and run length start to be affected and the maximum motor density that could be achieved at the same PEM12 buffer conditions. For kinesin-II we show here that velocity and run length start to decrease at a density of ~200 motors/μm, while this was at a density of ~30 motors/μm for OSM-3 and ~2 motors/μm for kinesin-I [7]. Furthermore, for kinesin-II, we find that the maximum density is ~500 motors/μm, while that was ~300 motors/μm for OSM-3 and ~20 motors/μm for kinesin-1 [7]. The effect of crowding decreases for all three motors at higher ionic-strength buffer conditions. For kinesin-II, no crowding effects could be observed in PEM80 (S1C Fig). This comparison shows that the different motor proteins appear to adapt vastly differently to crowded condition, with kinesin-II being least affected, substantially less so than OSM-3, which is from the same, kinesin-2, family of kinesins.

While sidestepping and torque-generation mechanism have been used to explain the how motors can overcome obstacles without significantly reducing the speed [14–17] such mechanisms cannot explain the behavior we have observed at high densities. At high densities, a significant portion of the microtubule lanes will be occupied (up to 13) with local motor densities higher than 50%. Under these conditions, sidestepping will hardly occur, since neighboring lanes are occupied most of the time. Following this reasoning, sidestepping does not affect our results for velocity and run length to a significant extend, at high densities. On the other hand, our findings indicate that kinesin-II can move effectively at high densities, in a ‘march-past’ fashion. This might include synchronization of the stepping of individual motors within the ensemble, but further studies will be required to verify this. What are the specific motor properties that allow kinesin-II to be far less affected by crowding than the other kinesins? We note here that the kinesin-II construct we have used here is not full length, but contains the KLP11/20 motor domains, attached to the well-defined (and in *E*. *coli* well expressed) stalk of *Drosophila* kinesin-1. Although the motility parameters of this construct appear very similar to that of full-length kinesin-II (which is very difficult to express in *E*. *coli*), we cannot exclude that the results would be different for the full-length construct. In contrast to kinesin-1 (human and *Drosophila*) homologues, whose chemomechanical cycle has been studied extensively [24,26–29], the direct measurements of the stepping cycle of OSM-3 and kinesin-II (KLP11/20) have not been performed yet. However, fundamental properties of single full-length KLP11/20 from *C*. *elegans* have previously been studied in optical tweezers and Total Internal Reflection Fluorescence (TIRF) microscopy-based assays [13,15,25]. More detailed studies have been performed on their mammalian homologues, KIF3A/B and KIF17. Studies on KIF3A/B indicate that the gating mechanism, which synchronizes the activity between the two different motor domains during stepping, is different in heterotrimeric kinesin-2 motors than in kinesin-1 [30–34]. Optical tweezers studies have demonstrated that the velocity of KIF3A/B is less affected by external force than kinesin-1, but that its run length decays rapidly with load. In addition, KIF3A/B was shown to possess fast detachment and reattachment kinetics, resulting in slipping [32]. Reattachment was shown to be almost 4-fold faster than that for Kinesin-1 [33]. In a recent study on KIF3A/B [34], it has been suggested that the anterograde IFT velocity is tuned either by regulating the attachment of KIF3A/B motors to IFT trains or modulating the rate of KIF3A/B binding to microtubules via intracellular obstacles. These findings indicate that KIF3A/B spends a larger portion of its hydrolysis cycle in a state with low affinity for microtubules, which could allow this motor to efficiently navigate crowded microtubule tracks full of obstacles, without affecting the motor’s overall run length.

Our findings provide important insights in the functional specialization of *C*. *elegans* heterotrimeric kinesin-II to deal with specific transport challenges. In the initial segments of *C*. *elegans* chemosensory cilia, large teams of kinesin-II (up to ~50) together drive the transport IFT trains. Further along the cilia the trains are gradually handed over to teams of (up to ~30) homodimeric OSM-3 motors moving in the same direction at higher velocity [1]. Our results suggest that both motors, kinesin-II and OSM-3, are adapted to work efficiently at small spacing, but kinesin-II to a substantially larger extent. This might be required for teams of kinesin-II to efficiently drive IFT along the highly crowded transition zone, which separates the ciliary lumen from the rest of the cell [1]. In the transition zone, the microtubules and their surroundings are not only crowded by a relatively high density of motor proteins, but also by static (protein) structures, such as the Y-shaped linkers. An interesting direction of future research might be to unravel to what extent kinesin-II’s specific ability of being able to cope well with high densities of dynamic crowders (i.e. other motor proteins), as we have identified here, is connected to its ability to cope with static crowders. In conclusion, our quantitative approach sheds new light on an important aspect of motor-driven transport: how motor protein function is affected by traffic jams.

## Supporting information

S1 Fig The effect of crowding on kinesin-II motility parameters at higher salt conditions (PEM80). A. Motor density as a function of kinesin-II concentration. B. Velocity, and C. run length as a function of kinesin-II density. Blue diamonds: parameter determinations on individual microtubule segments; red symbols: averages and standard deviations of multiple run length determinations within logarithmically scaled density intervals. At higher salt conditions (PEM80), ~10-fold shorter average run lengths (0.12 ± 0.03 μm) were obtained than at lower salt (Fig 4C), highlighting the strong salt dependence of kinesin-II microtubule affinity.
(TIF)

S1 Data Data of Figs 1, 3 and 4 in Microscoft Excel format. (XLSX)
