# Supplementary figures and images for: The crowding dynamics of the motor protein kinesin-II
Source: PLoS One. 2020 Feb 13;15(2):e0228930. doi: 10.1371/journal.pone.0228930 (PMC7018031; doi:10.1371/journal.pone.0228930)

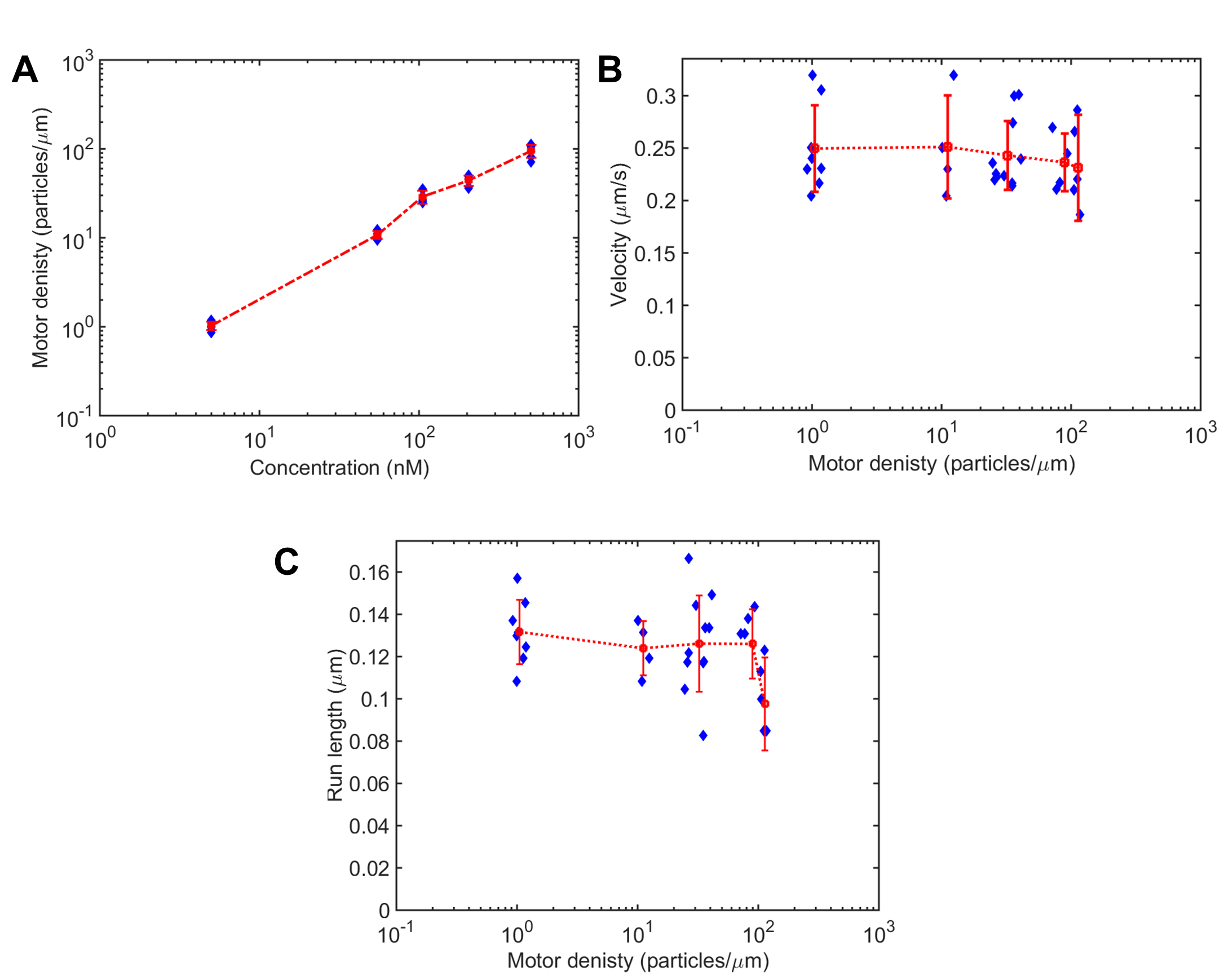

Supplement: S1 Fig — A. Motor density as a function of kinesin-II concentration. B. Velocity, and C. run length as a function of kinesin-II density. Blue diamonds: parameter determinations on individual microtubule segments; red symbols: averages and standard deviations of multiple run length determinations within logarithmically scaled density intervals. At higher salt conditions (PEM80), ~10-fold shorter average run lengths (0.12 ± 0.03 μm) were obtained than at lower salt (Fig 4C), highlighting the strong salt dependence of kinesin-II microtubule affinity. (TIF) [file pone.0228930.s001.tif]
